# Supplementary material for: Identifying relationships between imaging phenotypes and lung cancer-related mutation status: EGFR and KRAS
Source: Sci Rep. 2020 Feb 27;10:3625. doi: 10.1038/s41598-020-60202-3 (PMC7046701; doi:10.1038/s41598-020-60202-3)
Supplement: Supplementary file 1 — Supplementary Information. [file 41598_2020_60202_MOESM1_ESM.pdf]

	Radiomics		Semantic	
Number of Patients	116	114	158	157
**Clinical Features**	**EGFR (%)**	**KRAS (%)**	**EGFR (%)**	**KRAS (%)**
**Gender**				
Female	24.1	23.7	36.1	35.0
Male	75.9	76.3	63.9	80.3
**Smoking Status**				
Non-smoker	13.8	14.0	22.8	24.2
Current	19.8	20.2	15.2	14.6
Former	66.4	65.8	62.0	61.1

Table 1. Distribution of the clinical data used for EGFR and KRAS mutation status prediction experiments.

**Semantic Features**	**Nodule/Non-nodule**	**EGFR (%)**	**KRAS (%)**
**Axial Location**	Non-nodule		
Central		21.7	20.9
Peripheral		78.3	79.1
**Nodule Attenuation**	Nodule		
Solid		56.1	57.6
Partially solid		40.8	39.2
Ground glass		3.2	3.2
**Nodule Margins Dominant Pattern**	Nodule		
Irregular		17.2	15.2
Lobulated		30.6	32.2
Spiculated		25.5	26.6
Poorly defined		16.6	15.8
Smooth		10.2	10.1
**Nodule Margins Secondary Pattern**	Nodule		
Irregular		37.6	39.9
Lobulated		31.2	31.0
Spiculated		3.8	3.2
Poorly defined		16.6	15.2
Smooth		10.8	10.8
**Nodule Associated Findings**	Nodule		
Pleural retraction		10.2	10.1
Vascular convergence		20.4	20.3
Septal thickening		18.5	21.5
None		9.6	8.9
Attachment to pleura		7.6	7.6
Entering airway		21.7	20.3
Attachement to vessel		9.6	8.9
Bronchovascular bundle		2.5	2.5
**Centrilobular Nodules - Diffuse**	Non-nodule		
Absent		87.3	88.6
Present		12.7	11.4
**Nodule shape**	Nodule		
Complex		58.0	57.0
Round		29.3	29.7
Oval		12.1	12.7
Polygonal		0.6	0.6
**Nodule calcification**	Nodule		
Peripheral		6.4	7.0
None		93.6	93.0

**Semantic Features**	**Nodule/Non-nodule**	**EGFR (%)**	**KRAS (%)**
**Satellite Nodules in Dominant Lesion Lobe (>4mm noncalcified)**	Non-nodule		
Absent		77.1	75.9
Solid		7.6	8.2
Partially-solid		5.1	5.1
Non-solid		10.2	10.8
**Nodules in Non-Lesion Lobe Same Lung (>4mm noncalcified)**	Non-nodule		
Absent		82.8	81.6
Solid		5.7	6.3
Partially-solid		1.9	2.5
Non-solid		9.6	9.5
**Nodules in Contralateral Lung (>4mm noncalcified)**	Non-nodule		
Absent		76.4	75.3
Solid		5.1	5.7
Partially-solid		7.6	7.6
Non-solid		10.8	11.4
**Nodule Internal Features**	Nodule		
Not applicable		50.3	51.3
Cavitated		8.9	8.2
Internal air bronchogram sign		28.0	27.8
Reticulation		7.0	7.0
Necrosis		3.2	3.2
Nodule cysts		2.5	2.5
**Nodule Periphery**	Nodule		
Emphysema		26.1	26.6
Normal		70.1	69.6
Fibrosis		3.8	3.8
**Emphysema**	Non-nodule		
absent		47.8	48.7
present		52.2	51.3
**Fibrosis**	Non-nodule		
absent		89.8	88.6
present		10.2	11.4
**Lung Parenchyma Features**	Non-nodule		
Bronchial wall thickening		33.8	32.9
Tree-in-bud sign		1.9	2.5
Airway ectasia		9.6	9.5
Mosaic oligemia		5.1	5.1
Bronchial prominence		0.6	1.3
Bronchiectasis		3.8	4.4
Normal		45.2	44.3
**Dominant Emphysema Pattern**	Non-nodule		
Paraseptal		12.7	12.0
Centrilobular		37.6	38.0
Panacinar		1.9	1.3
Not applicable		47.8	48.7
**Secondary Emphysema Pattern**	Non-nodule		
Paraseptal		17.2	17.1
Centrilobular		8.3	7.6
Panacinar		0.6	0.6
Not applicable		73.9	74.7

Table 2. Distribution of the semantic data used for EGFR and KRAS mutation status prediction experiments.

Semantic Feature	Score
Emphysema: Present	0.2076 +/- 0.0648
Nodule Periphery: Normal	0.1492 +/- 0.1027
Lung Parencyma Features: Normal	0.1159 +/- 0.0498
Nodule Attenuation: Solid	0.0480 +/- 0.0287
Nodule Attenuation: Partial solid	0.0470 +/- 0.0191
Smoking status: Nonsmoker	0.0425 +/- 0.0181
Nodule Internal Features: Internal air bronchogram sign	0.0423 +/- 0.0241
Gender: Male	0.0421 +/- 0.0167
Nodule Shape: Round	0.0392 +/- 0.0199
Axial Location: Peripheral	0.0333 +/- 0.0329
Nodule Margins-Secondary Pattern: Lobulated	0.0294 +/- 0.0238
Nodule Margins-Dominant Pattern: Lobulated	0.0275 +/- 0.0230
Nodule Associated Findings: Septal thickening	0.0275 +/- 0.0169
Smoking status: Former	0.0262 +/- 0.0114
Nodule Margins-Dominant Pattern: Poorly defined	0.0274 +/- 0.0238
Nodule Margins-Dominant Pattern: Spiculated	0.0202 +/- 0.0173
Nodule Associated Findings: Entering airway	0.0187 +/- 0.0194
Nodule Margins-Secondary Pattern: Poorly defined	0.0104 +/- 0.0215
Nodule Associated Findings: Vascular convergence	0.0085 +/- 0.0135
Centrilobular Nodules: Diffuse	0.0069 +/- 0.0195
Fibrosis: Present - Diffuse	0.0069 +/- 0.0155
Nodule Margins-Dominant Pattern: Smooth	0.0060 +/- 0.0248
Dominant Emphysema Pattern: Paraseptal	0.0054 +/- 0.0200
Satellite Nodules in Dominant Lesion Lobe (greater than 4mm noncalcified): Non-solid	0.0028 +/- 0.0086
Nodules in Contralateral Lung (gretater than 4mm noncalcified): Non-solid	0.0026 +/- 0.0082
Nodules in Non-Lesion Lobe Same Lung (greater than 4mm noncalcified): Non-solid	0.0025 +/- 0.0074
Secondary Emmphysema Pattern: Paraseptal	0.0019 +/- 0.000
Nodule Margins-Secondary Pattern: Smooth	0.0010 +/- 0.0049
Nodule Shape: Oval	0.0010 +/- 0.0046
Nodule Associated Findings: Attachment to vessel	0.0010 +/- 0.0055
Nodule Calcification: Peripheral	0.0008 +/- 0.0063
Nodule Associated Findings: Pleural retraction	0.0007 +/- 0.0034
Nodules in Non-Lesion Lobe Same Lung (greater than 4mm noncalcified): Solid	0.0005 +/- 0.0032
Nodule Associated Findings: None	0.0002 +/- 0.0020
Satellite Nodules in Dominant Lesion Lobe (greater than 4mm noncalcified): Partially solid	0.0002 +/- 0.0018
Nodules in Contralateral Lung (gretater than 4mm noncalcified): Solid	0.0002 +/- 0.0018
Nodule Internal Features: Reticulation	0.0002 +/- 0.0020

Table 3. Semantic features importance scores.

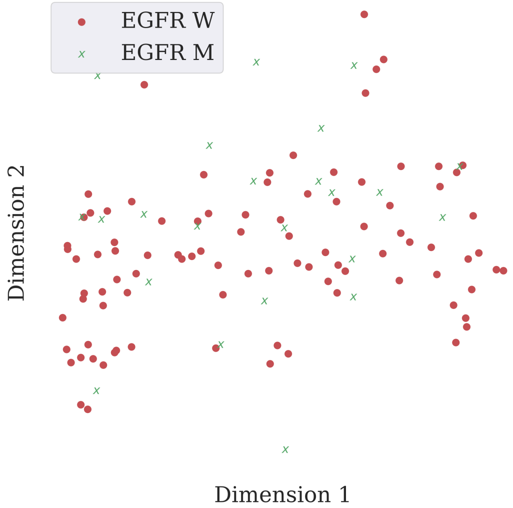

(a) EGFR t-SNE using radiomic features.

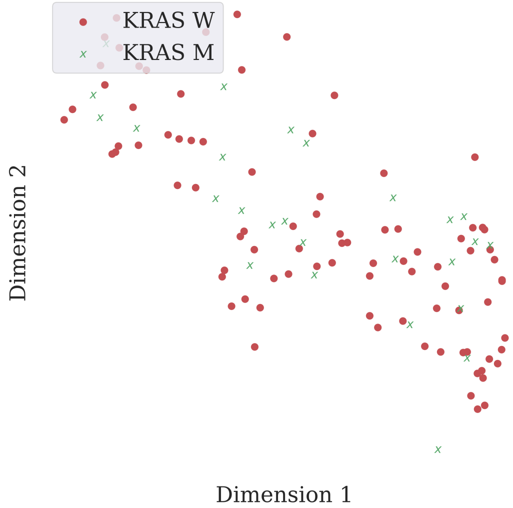

(b) KRAS t-SNE using radiomic features.

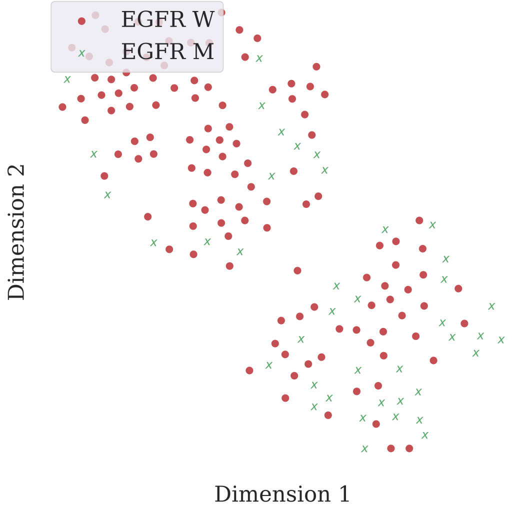

(c) EGFR t-SNE using semantic nodule features.

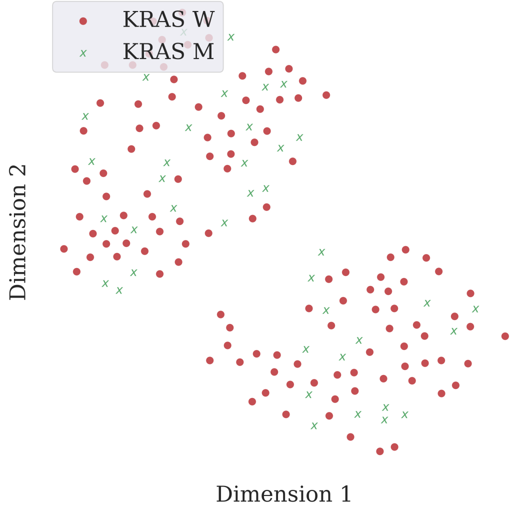

(d) KRAS t-SNE using semantic nodule features.

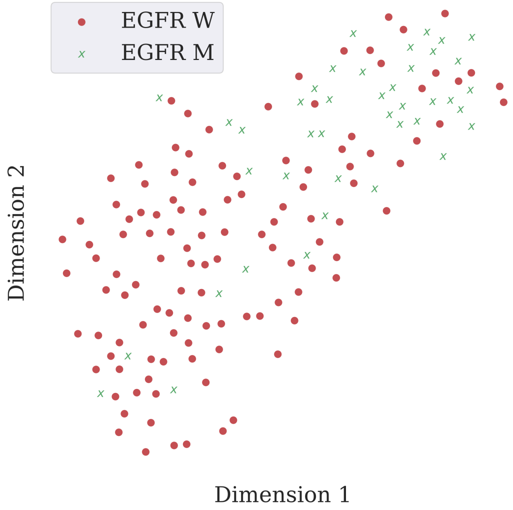

(e) EGFR t-SNE using semantic non-nodule features.

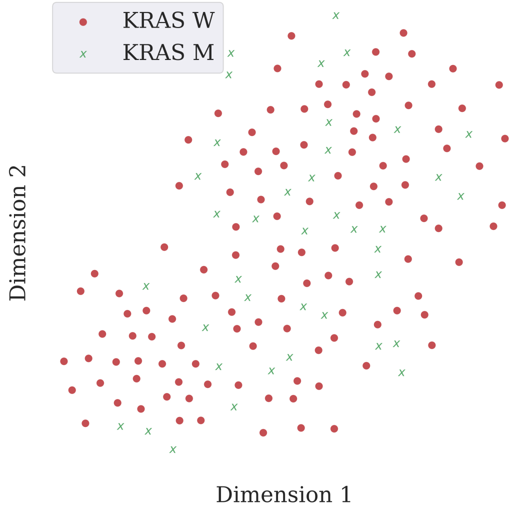

(f) KRAS t-SNE using semantic non-nodule features.

Figure 1. Visualisation of sample distributions based on PCA and t-SNE. Each point is coloured according to its mutation status, with red and green dots representing wild type and mutated cases, respectively.
